# Design and Implementation of Brief Interventions to Address Noncommunicable Diseases in Uzbekistan

**DOI:** 10.9745/GHSP-D-23-00443

**Published:** 2024-08-27

**Authors:** Olakunle Alonge, Maysam Homsi, Mahnoor Syeda Rizvi, Regina Malykh, Karin Geffert, Nazokat Kasymova, Nurshaim Tilenbaeva, Lola Isakova, Maria Kushubakova, Dilbar Mavlyanova, Tursun Mamyrbaeva, Marina Duishenkulova, Adriana Pinedo, Olga Andreeva, Kremlin Wickramasinghe

**Affiliations:** a University of Alabama at Birmingham, Birmingham, AL, USA.; b Johns Hopkins Bloomberg School of Public Health, Baltimore, MD, USA.; c World Health Organization Regional Office for Europe, Copenhagen, Denmark.; d World Health Organization Country Office, Tashkent, Uzbekistan.; e World Health Organization Country Office, Bishkek, Kyrgyzstan.; f Research Institute of Sanitation, Hygiene and Occupational Diseases, Ministry of Health of the Republic of Uzbekistan, Tashkent, Uzbekistan.; g Department of Disease Prevention and State Epidemiological Surveillance, Ministry of Health of Kyrgyzstan, Bishkek, Kyrgyzstan.; h Tashkent Pediatric Medical Institute, Tashkent, Uzbekistan.; i Kyrgyz State Medical Academy, Bishkek, Kyrgyzstan.; j Republican Center of Health Promotion and Mass Communication under Ministry of Health, Bishkek, Kyrgyzstan.

## Abstract

In Uzbekistan, NCDs, including cardiovascular diseases, cancer, and diabetes, accounted for over 80% of mortality in 2019. In 2021, national stakeholders, in conjunction with the World Health Organization, identified brief interventions (BIs) to implement in primary health care settings to change unhealthy behaviors and reduce the burden of NCDs in the country. BIs consist of a validated set of questions to identify and measure NCD behavioral risk factors and a short conversation with patients/clients about their behavior, as well as the provision of a referral opportunity for further in-depth counseling or treatment if needed. We used a multimethod approach of document review, participatory workshops, and key informant interviews to describe how BIs were designed and implemented in Uzbekistan and generated a theory of change for its large-scale implementation. BIs in Uzbekistan targeted 4 risk factors (alcohol use, tobacco use, unhealthy diet, and physical inactivity) and entailed training clinicians on how to conduct behavioral change counseling using the 5As and 5Rs toolkit, conducting supportive supervision, and using feedback to improve service delivery. The program was collaboratively designed by multiple stakeholders across sectors, including the Ministries of Health, Higher Education, Science, and Innovations, with buy-in from key political leaders. The potential impact of the program (i.e., reducing the incidence of NCDs) was mediated by several intermediate and implementation outcomes at the individual, primary care, and community levels operating along multiple pathways. Significant health system challenges remain to the program, such as limited human resources, lack of incentives for clinicians, outdated systems and data collection processes for performance monitoring, and coordination among different relevant sectors. These and other challenges will need to be addressed to ensure the effective large-scale implementation of BIs in Uzbekistan and similar LMICs.

## Abstract (precis)

Large-scale implementation of brief interventions to address behavioral risk factors of noncommunicable diseases in primary care health settings in Uzbekistan is limited by a lack of human resources, a supportive system, and clear incentives for clinicians.

## Key Messages

- Brief interventions (BIs), implemented by clinicians in primary health care settings, are ways to address the growing burden of noncommunicable diseases (NCDs) in low- and middle-income countries.
- Pathways for the large-scale implementation of BIs in Uzbekistan include key strategies (e.g., supportive supervision and audit feedback), implementation outcomes (e.g., acceptability of the interventions by different stakeholders), and intermediate outcomes (e.g., changes to clinicians’ knowledge and self-efficacy to practice counseling for NCD risk factors).
- To achieve large-scale effectiveness with BIs, policymakers and program managers should consider key health system challenges, including shortages of human resources for health, clinician incentives, improvements to data systems and use, and performance monitoring.

## BACKGROUND

Noncommunicable diseases (NCDs), including cardiovascular diseases (CVDs), diabetes, and cancers, are the leading cause of premature deaths in Uzbekistan, accounting for over 80% of all mortality in 2019.^1^^,^^2^ CVDs alone accounted for most of these deaths, and over 40% of the adult population (ages 30–79 years) have hypertension in the country.^3^^,^^4^ Behavioral risk factors for CVDs and most other types of NCDs include alcohol consumption, tobacco use, physical inactivity, and unhealthy diet.^5^

About 26% of youths and adults aged 15 years and older in Uzbekistan are current alcohol users (i.e., drank alcohol in the past 30 days).^6^ In 2016, the prevalence of heavy episodic drinking (i.e., consuming 60 g or more of pure alcohol on at least 1 occasion in the past 30 days) in the population aged 15 years and older was 8.1% in Uzbekistan (males, 14.6%; females, 2.0%).^6^ The prevalence of heavy episodic drinking is likely to have further increased because of the COVID-19 pandemic in 2020–2023.^7^

According to the 2021 World Health Organization’s (WHO) report on the global tobacco epidemic, the prevalence of current tobacco use among adults aged 15 years and older in Uzbekistan was 18.1% (males, 35.1%; females, 1.2%).^8^ About 13.9%–19.1% of adults, especially women and those living in urban areas, are physically inactive (i.e., engaging in less than 150 minutes per week of moderate-intensity physical activity) in both countries.^9^ Unhealthy diet such as excessive salt intake is also common.^3^^,^^10^

To address this huge burden of NCDs and their risk factors, in 2021, national stakeholders, in concert with the WHO European Office for the Prevention and Control of NCDs (WHO NCD Office), selected brief interventions (BIs) as a major approach for addressing the burden of NCDs. The selection process was in line with the WHO Global Action Plan for the Prevention and Control of Non-communicable Diseases 2013–2020^11^ and was informed by the WHO Best Buys interventions for NCD control.^12^

BIs are a set of intervention components usually implemented in, but not limited to, primary health care (PHC) settings to initiate change in an unhealthy behavior (e.g., quit smoking, quit or reduce alcohol use, improve diet, and increase physical activity).^13^^–^^15^ They usually consist of 2 intervention components: (1) a set of questions to identify a patient/client’s pattern of unhealthy behavior and measure NCD risk factors (e.g., the Alcohol Use Disorders Identification Test for alcohol drinking, the Global Physical Activity Questionnaire for lack of physical activity); and (2) a brief conversation with patients/clients about their behavior, as well as referral for further in-depth counseling or treatment if needed.^16^^–^^18^ The questions included as part of BIs have been previously established as valid tools for identifying patterns of unhealthy behaviors through psychometric and correlation studies among populations in several countries.^19^^–^^22^ BIs are premised on the generalized theory that people may change their behavior with appropriate assistance and effort, and an offer of help to change is more motivating than the advice to do so.^23^

BIs consist of questions to identify patterns of unhealthy behaviors and a conversation about those behaviors to initiate behavior change.

Whereas BIs have been shown to increase quit attempts for smoking in other countries,^23^ their effectiveness in Uzbekistan and other Central Asian countries has not been established. Moreover, the adoption of BIs in Uzbekistan will not achieve impact at the population level without their effective implementation.^24^ Pathways to effective implementation are influenced by the contexts, including the availability of resources and support systems; factors that can be both facilitators and barriers to achieving impact; and dynamic interactions among the intervention and the context,^24^ which may call for different implementation strategies during the different stages of implementation.^24^ However, there are gaps in our understanding of the implementation pathways and theories for supporting the effective implementation of BIs in LMICs.^24^^,^^25^ Similarly, there are gaps in our knowledge of how to implement complex interventions (like BIs) that require multisectoral action to address the growing burden of NCDs.

We aim to describe a multicountry and multisectoral collaborative process for designing and implementing BIs for NCD prevention and control in the Syrdarya region, Uzbekistan. The pathways and theories identified through this process directly address the knowledge gaps on how to implement BIs effectively in Uzbekistan and provide relevant implementation strategies and outcomes for more effective implementation of BIs in Uzbekistan and other LMICs.

## METHODS

### Implementation Setting

The BI program was implemented in Uzbekistan in the Syrdarya region, which was chosen as an early site for the BI program because it encompasses both urban and rural populations and is the pilot region for the new health system reform in Uzbekistan focused on strengthening health care financing and PHC delivery.^26^ It is in the central part of the country and has an estimated population of 896,600 in 2023, with a low-income population of 12.3% compared to the country’s average of 11.5%.^27^ While subnational statistics on the prevalence of NCDs and related behavioral risk factors are mostly unavailable, Syrdarya region has an incidence rate of obesity of 31.3 per 100,000 population compared to the country’s average of 408.3 per 100,000 population.^27^ Like the rest of the country, the state-run health care delivery system in Syrdarya is mainly organized around hospitals, polyclinics (multidisciplinary outpatient clinics), and rural family physician posts.^28^^,^^29^ As part of the health system reform, newly established multidisciplinary PHC teams have replaced a third of the PHC providers in PHC facilities (encompassing polyclinics and rural family physician posts) in Syrdarya region since 2021.^26^ Each PHC team is assigned to 2,000 people and comprises 1 family doctor, 1 practicing nurse (similar to a nurse practitioner), and 2 patronage nurses (who conduct regular home visits), with 1 midwife shared among 2–3 teams.^26^

There are plans to eventually implement the program in all 13 regions of Uzbekistan and the capital city of Tashkent in 2024 and onward.

### Data Collection

**FIGURE 1:**
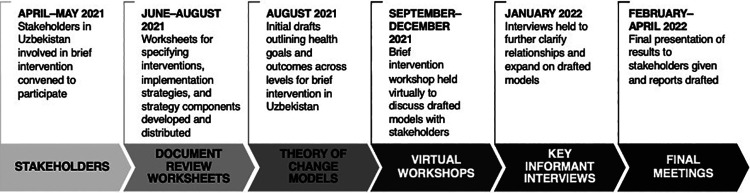
Timeline of Workshop Meetings and Model Development for Brief Interventions in Uzbekistan

We combined document review, participatory theory of change workshops, and key informant interviews to describe the design and large-scale implementation of BIs in Uzbekistan and generate theories of change for how BIs could potentially lead to population-level impact in the Uzbekistan context (Figure 1). Thereafter, we convened a multistakeholder regional meeting to validate and contextualize the theories across the Central Asian region. A theory of change outlines the relationships among a set of outcomes that must be fulfilled for a program goal to be achieved—it makes explicit the assumptions under which the outcomes are obtained and the contextual factors that influence the relationships among these outcomes.^30^^,^^31^ The methods applied in this study follow a similar approach used for developing pathways and theories for large-scale implementation of school-based mental health programs.^32^

First, 11 relevant stakeholders involved with BIs were identified and convened between April 2021 and April 2022 to describe how the interventions were designed and initially implemented and codevelop theories of change for their large-scale implementation in Uzbekistan. The stakeholder identification was led by the WHO NCD Office and facilitated by the WHO Country Office, which had been working closely with various national and subnational institutions and actors to support the planning and implementation of BIs in Uzbekistan (Table 1). The identified stakeholders were part of a bigger stakeholder group convened by the WHO NCD Office to examine theories of change for NCD programs, including BIs and school nutrition policies, in Central Asia. This bigger stakeholder group included 48 stakeholders from the health, education, food, and agriculture sectors across Uzbekistan and Kyrgyzstan.

**TABLE 1.** Stakeholder Composition for Brief Interventions to Address Noncommunicable Diseases in Uzbekistan

**Agency**	**Role/Position and Level**
Ministry of Health	
Department on Prevention and Treatment care (Noncommunicable Diseases sector)	Deputy Head/
Centre for Supporting a Healthy Lifestyle and Increasing Physical Activity of the Population	Senior Specialist
Centre for Professional Development of Medical Workers, Department of Food Hygiene	Professor
Tashkent Medical Academy, Department of General Practice	Professor
State Health Insurance Fund under the Cabinet of Ministries	Senior Specialist
Tashkent Paediatric Medical Institute	
Department of General Practice	Associate Professor, Member of National Association of General Practitioners
	Senior Lecturer
	Researcher
	Senior Teacher
Central District Multi-profile policlinic/District Medical Unit, Boyavut district, Syrdarya region	Doctor
World Health Organization Country Office	Technical Officer, National Professional Officer on Noncommunicable diseases

Second, a document review worksheet was developed using Proctor’s framework for specifying implementation strategies^33^ and the Consolidated Framework on Implementation Research (CFIR)^34^ to extract information on the intervention/strategy components included as part of the BIs and the process, settings, and activities involved in their implementation (Supplement). The worksheet was completed by the stakeholders (Table 1), drawing on their experience with the design and implementation of BIs in Uzbekistan and information from accessible policy documents and data. About 20 government documents (including decrees, regulations, and orders) and data sources were consulted for completing the BI worksheet.

Third, a description of the various activities included as part of the BIs and their implementation processes was compiled by the study team based on the information extracted from the worksheet. The relationships among these activities and different outcomes, as described in the worksheet, were also used to create initial drafts of the theory of change (TOC) model showing the pathway linking the interventions to population-level health impact. The TOC model was developed by listing and sequencing the relevant overall health goals and outcomes at various levels (individual, community, PHC settings) linked to the interventions and the policy advocacy activities that supported the adoption of the interventions. This approach followed the convention for developing TOC described elsewhere.^30^

Fourth, a participatory virtual workshop, about 90 minutes long, was organized with the relevant stakeholders to further develop and validate the initial drafts of the TOC model for the Uzbekistan context. The model-building activities were conducted using Miro software, which allowed the study team to edit the model and take notes as they were being discussed by the stakeholders in real time. The workshop discussion focused on clarifying the specified outcomes and the relationships among the outcomes, facilitators and barriers of the interventions, and any context-specific implementation strategies that may have been deployed, drawing upon the ongoing and real-life implementation of the interventions. Following the workshop, a key informant interview was conducted with the main technical person at the WHO Country Office to clarify any follow-up questions on the relationships as specified in the TOC.

A final dissemination meeting was organized with the WHO NCD Office, WHO Country Offices, and key stakeholders from Uzbekistan and other countries from the WHO European Region, including the bigger stakeholder group, to review and validate the final drafts of the TOC and contextualize it for the region (i.e., qualitatively evaluate whether the relationships, challenges, and strategies described are relevant for other similar countries). All workshops, meetings, and interviews were conducted in English and Russian with simultaneous interpretation.

## BRIEF INTERVENTIONS TO ADDRESS BEHAVIORAL RISK FACTORS OF NONCOMMUNICABLE DISEASES IN UZBEKISTAN

BIs in Uzbekistan implemented at the PHC level targeted 4 risk factors (alcohol use, tobacco use, unhealthy diet, and physical inactivity). The main intervention strategies included training clinicians to conduct behavioral change counseling using the 5As and 5Rs toolkit,^35^^–^^37^ conducting supportive supervision, and using interactions during supportive supervision to provide feedback for improving service delivery. The clinician training also covered how to implement CVD risk assessment and stratification for patients aged 40 years and older attending primary care visits based on the WHO package of essential NCD interventions protocol^38^ and HEARTS technical protocol for CVD management in primary care.^39^

BIs in Uzbekistan implemented at the PHC level targeted 4 NCD risk factors: alcohol use, tobacco use, unhealthy diet, and physical inactivity.

The BI implementation involved activities at different levels. At the national level, a federal agency managing the Uzbekistan State Health Insurance Fund (which was previously under the Cabinet of Ministries before 2024, the Ministry of Health from 2024, and expected to become autonomous from 2027) in conjunction with the Centre for the Development of Professional Qualification of Medical Workers (a federal institution responsible for training and introduction of modern medical technology for NCD prevention and control nationally) was responsible for planning the program. These agencies designed the BI training curriculum with expert support from the WHO NCD Office and approvals from the Ministry of Health and its Department of Education and Science in Uzbekistan. At the regional level, the health commissioners and chief experts from the Regional Health Department conducted training and supervisory activities for care providers at the facility level. Training was also provided to medical post-graduates about to enter the health workforce. At the PHC facility level, trained chief doctors, heads of departments, PHC teams, and other physicians and nurses provided BIs directly to clients.

In 2021, a training of trainers’ workshop was conducted over 5 days for 14 health care professionals (comprising chief experts in endocrinology, radiology, internal medicine, primary care physicians, and nurses) at the regional level. The trainers then cascaded training to the care providers at the PHC level via in-person workshops conducted over 2–3 days. As of February 2023, a total of 350 health care providers (77 primary care physicians and 273 nurses) had been trained throughout the region, and there are ongoing plans to train another 325 health care providers (46 primary care physicians and 279 nurses).

The trained care providers delivered the BI assessment and counseling to clients during primary care visits as in-person interviews lasting 3–20 minutes. Trained chief experts from the Regional Health Department conducted supervisory visits to the PHC facilities monthly. During the visits, they observed the BI implementation and reviewed program records. They also provided real-time feedback to the trained health care providers on their program delivery.^40^^,^^41^

### Theory of Change and Implementation Outcomes

**FIGURE 2:**
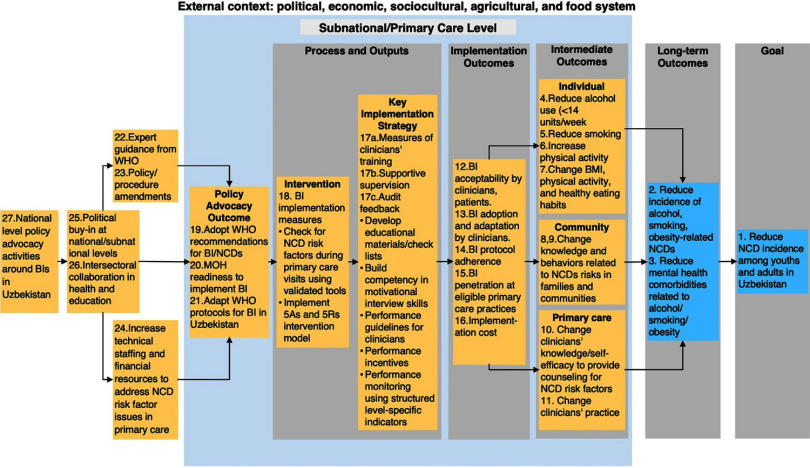
Theory of Change for Brief Intervention Implementation in Uzbekistan^a^ Abbreviations: BI, brief intervention; MOH, Ministry of Health; NCD, noncommunicable disease; WHO, World Health Organization. ^a^ Key assumptions: (1) National MOHs and other stakeholders willing to prioritize BI, (2) availability and motivation of clinicians to implement BI, (3) most youths and adults in Uzbekistan attend primary care visits at least once a year, and (4) others. Yellow boxes are preconditions and numbers indicate stakeholders’ relative importance ranking of preconditions/outcomes needed to achieve the goal, with 1 being the most important.

Figure 2 shows the TOC for BI implementation in Uzbekistan. BIs were being implemented to reduce the incidence of NCDs among youth and adults by specifically targeting a reduction in the incidence of CVDs, stroke, diabetes, cancers, and mental health disorders associated with the use of alcohol and smoking among this population. The BI program universally targeted youths and adults aged 15 years and older, irrespective of gender and socioeconomic status, who presented in PHC settings for health services, including mobile health services provided by multidisciplinary PHC teams. The BIs and their implementation strategies (mainly training of clinicians, supportive supervision, and audit feedback provided to trained clinicians) (Table 2) were anticipated to lead to the following intermediate outcomes: (1) PHC level: clinicians’ increased knowledge and self-efficacy to provide counseling for reducing exposure to NCD risk factors; (2) individual-level: patients and client reduction in alcohol use and smoking and increased healthy eating habits and physical activity adherence; and (3) community-level: changes in knowledge and behaviors among family members and network of patients and clients that received BIs (Figure 2).

**TABLE 2.** Key Implementation Strategies for Brief Interventions to Address Noncommunicable Diseases in Uzbekistan

**Key Implementation Strategies**	**Implementation Challenges That Strategies Respond to**	**Supportive System Needed for Strategies to Work**
Develop brief intervention training curriculum for clinicians in primary health care.	Lack of a standardized approach for brief intervention training among clinicians and healthcare providers.	Funding support and interministerial coordination for training and service delivery.
Use a training of trainers’ approach to cascade training to Regional Health Department and primary health care teams.	Limited human resources for training. Structural barriers to rapid dissemination of training.	Human, financial, and technical support from the Regional Health Department.
Conduct structured, onsite, in-person interviews for brief interventions at primary health care facilities.	Limited behavioral counseling during provider-client interactions.	Functional and conducive facility environment. Well-maintained referral systems and mechanism for service coordination.
Provide supportive supervision to monitor progress of trained clinicians.	Variable program implementation fidelity, and possible declining quality of brief intervention implementation.	Systems for incentivizing and/or motivating staff.
Provide audit feedback based on structured indicators.	Data collection conducted on paper; inaccurate data entry / loss of data.	Development of information systems for better data collection, management, and analyses.

These intermediate outcomes were mediated by implementation outcomes, including acceptability of the BI to clinicians, patients, and their family members, adoption of the BI by the clinicians, and implementation fidelity to the WHO protocols for conducting the BI along with any systematic adaptation, and the spread of BIs within PHC practices. The decision to implement the BI and its implementation strategies across primary care in Uzbekistan was based on high-level advocacy with the government of Uzbekistan by different partners working to address NCDs in the country coupled with technical support from the WHO, which created political buy-in and facilitated interministerial cooperation, development of policy guidelines, and increased resources for BI implementation.

During the TOC development exercise, critical gaps were uncovered in a rate-limiting step between the BI and implementation outcomes, and the implementation outcomes may not be achieved as the program was being implemented at the time of writing. For example, clinicians’ motivation to implement BIs (which may have been seen as an additional responsibility to their usual practice) varied significantly based on their workload, and it was not clear how the clinicians’ performance was assessed over time. Hence, additional implementation strategies would be required for the successful implementation of the BI. These implementation strategies may include developing and adopting clear performance guidelines and incentives for conducting BIs at PHC facilities, building clinician competency in motivational interview skills and quality improvement studies, and developing a monitoring plan to collect and track data on performance and outcomes.

Key assumptions that underlie the TOC for BI implementation in Uzbekistan include the willingness of relevant ministries and stakeholders to prioritize BI as we observed, availability of a sufficient number of clinicians in PHC settings to implement BIs, and positive health-seeking behavior by adults and youths visiting these PHC settings at least once a year for their annual physical and wellness assessments.

## DISCUSSION

The BI program in the Syrdarya region of Uzbekistan was the first large-scale BI implementation program in Central Asia. Previous pilot projects have been conducted in Kazakhstan but did not cover an entire region and were not sustained due largely to a lack of training and adequate resources.^42^ The BI program in Syrdarya successfully designed a professional curriculum for BI training and cascade training to health care providers at PHC facilities throughout the entire region within the space of 2 years while actively integrating the BI program into the existing health care delivery system to ensure sustainability. This success may have been facilitated by 2 main factors. First, the program received political support from the highest level of government and was strategically embedded within a broader health system reform program in Uzbekistan with a widespread legislative mandate.^26^ Second, the program was championed by the Uzbekistan State Health Insurance Fund (under the Cabinet of Ministries), which has legislative power to serve as the single national pooling and health services purchasing agency in Uzbekistan.^26^ Hence, the program was successfully routinized as part of the health services arrangement for the Syrdarya region and leveraged state government funds and implementation apparatus at various levels. It is anticipated that the BI program will follow this pattern to scale nationally as part of the broader health system reform.

The BI program in Syrdarya successfully designed a professional curriculum for BI training to health care providers at PHC facilities while actively integrating the BI program into the existing health care delivery system to ensure sustainability.

The target population for the BI program included all youths and adults aged 15 years and older, irrespective of gender and socioeconomic status (i.e., a universal targeting approach). While this approach was likely to ensure access for all eligible individuals attending PHC for health services, it may worsen health inequities regarding NCD outcomes, risks, and related services if there are other cost barriers that preclude populations from a low socioeconomic class from seeking PHC services, as some studies may have suggested in Uzbekistan.^28^ Hence, as the BI program continues to be rolled out, it should be accompanied by other strategies that target the poor in Uzbekistan (e.g., performance-based health financing that targets the poor in Uzbekistan).^43^^,^^44^

The implementation pathway for BIs in Uzbekistan operated through various intermediate outcomes at different socioecological levels, including changes in knowledge, self-efficacy, and practice of clinicians to provide counseling for NCD risk factors at the primary care levels; changes in knowledge about NCD prevention and control among family members at the community level; and reduction in alcohol use and smoking at the individual level. Evidence from the literature suggests that these intermediate outcomes are plausible.^45^^–^^52^ Several studies have shown that training in behavioral counseling for BI leads to increased knowledge and self-efficacy among clinicians to deliver BIs,^47^^–^^49^ and these, in turn, are strongly associated with improvements in practice and health behavior change and maintenance.^45^^,^^50^ In 1 of the studies, BI training for tobacco smoking cessation was shown to have a sustained positive impact on practice among clinicians to advise, assess, and assist patients in tobacco smoking cessation and refer them for treatment.^49^ Studies have also shown that positive behavior changes related to tobacco smoking, physical inactivity, and being obese in individuals are associated with similar positive behavioral changes among their married partners.^51^ The change mechanisms by which family members influence one another are proposed to happen through family support, supervision, and modeling of healthy behaviors.^52^

The intermediate outcomes were mediated by implementation outcomes, including acceptability of the BI by clinicians, clients, and community members; adherence to the adapted BI protocol by clinicians; and spread of the BI program among primary practices in Uzbekistan. Implementation science theories and frameworks suggest that implementation outcomes around evidence-supported interventions, such as BIs deployed in health care settings, are good predictors of intermediate and health outcomes.^53^^,^^54^ However, there are limited studies that have been conducted to test these theories for BI programs, though guidelines exist on how to operationalize them for BI research.^55^^,^^56^ Some of the implementation strategies and support systems needed to facilitate these implementation outcomes are also not currently in place in Uzbekistan, and there are currently no tools or mechanisms to systematically collect data and assess changes with time on the implementation outcomes and other outcomes identified along the implementation pathways as specified in the theory of change. Further, some of the TOC key assumptions may need to be empirically tested to explain the impact pathways in Uzbekistan.

It is important to highlight that the BI program we described is still underway, so the TOC and implementation pathways for the program will still likely evolve. However, the TOCs, outcomes, strategies, and pathways described are relevant for initially guiding a systematic evaluation and learning on how to successfully implement a BI program at scale in Uzbekistan and provide lessons for similar countries in the Central Asian region and other LMICs.

There are several health system challenges that will need to be addressed for the BI program to achieve impact in Syrdarya; these challenges may also be relevant to any potential national scale-up program. Implementation of BIs was affected by a shortage of clinicians to directly provide counseling and handle the referrals that may follow and the increased workload caused by the insufficient number of clinicians in the health system. Some clinicians perceived BI implementation as an additional burden on top of their existing workload, which may further demotivate them in implementing the program effectively. The program was also limited by the fragmented information system and inconsistent data collection and analysis for performance monitoring. Data collection for tracking clinical services and patient outcomes is still being conducted on paper at many health facilities, resulting in inaccurate data entry and loss of data. Moreover, the data that are collected are not used to improve the program delivery because of a limited capacity in quality improvement practices within the health care delivery system. Further, there was a perception among clinicians and health providers that supportive supervision and data collected as part of the BI could be used punitively against them, and there was a general lack of incentives to facilitate the additional work burden that clinicians and health providers had to undertake as part of the BI implementation.

The implementation of BIs was affected by a shortage of clinicians to directly provide counseling and handle the referrals that may follow.

It is important for these health system challenges to be carefully considered and implementation strategies designed and tested to address them moving forward. These implementation strategies may include developing and adopting clear performance guidelines and incentives for conducting BIs at primary care facilities, building competency of clinicians in motivational interview skills and quality improvement studies, and developing a monitoring plan to collect and track data on performance and outcomes, including an electronic data system.

For next steps, the TOC will be used to design, package, and test selected implementation strategies for addressing gaps in the TOC, as well as to develop tools and mechanisms to systematically collect data and assess changes with time on the implementation outcomes and other outcomes identified along the implementation pathways. Further, the TOC will be used to iteratively guide the evolution of BIs and develop learning and capacity-building activities for implementers on implementation research and to guide how to strengthen program implementation and health services delivery more broadly.

### Strengths and Limitations

The TOC approach used in this study has been previously shown to be flexible and useful in accommodating multiple perspectives in studying complex programs and their implementation pathways, especially within the context of large-scale implementation or scale-up efforts.^31^^,^^32^ It is particularly useful in facilitating group learning, identifying critical gaps in the implementation pathways, and developing implementation strategies with key partners in a dynamic and iterative fashion. However, the TOC approach, as applied in this article, had some limitations. First, the TOC workshops were conducted virtually (due to COVID-19 pandemic restrictions), which may have limited the discussions around the program and its implementation pathways and the ability to form consensus on key outcomes. We tried to overcome this limitation by prepopulating the TOC diagrams based on the document synthesis ahead of the meetings so that the workshop could focus on validating the nature and forms of different preconditions within the TOC and the relationships among them. The logistics of virtually convening stakeholders for the TOC workshops during the COVID-19 pandemic also precluded the involvement of patients and others. However, several studies have shown a high level of participants’ satisfaction and acceptability of BIs among patients in diverse settings.^57^^–^^61^ Second, the key informant exit interview after the TOC workshops was limited, which may have skewed the emphasis on certain outcomes and relationships as described. However, we preserved the views from the workshops as much as possible and only used the interview to clarify statements and descriptions of concepts derived from the workshops.

## CONCLUSION

A BI program (encompassing training of clinicians, conducting supportive supervision, audit feedback and other implementation strategies) was successfully designed and implemented at scale in the Syrdarya region of Uzbekistan and embedded within the health care delivery system, leveraging an ongoing health system reform in the country. The pathways by which the program led to impact can be potentially mediated by several intermediate and implementation outcomes at different socioecological levels, including at the individual, PHC, and community levels. However, there are critical implementation and health system challenges that may hamper the effectiveness of the program in the region and any potential scale-up plans in Uzbekistan. The pathways, theories, and implementation research outcomes identified in this article will facilitate systematic learning and evaluation around BIs and NCD prevention and control programs in LMIC more broadly.

## Notes

**Peer Reviewed**

**First Published Online:** July 11, 2024.
